# Awareness Among Parents About Pneumococcal Conjugate Vaccine in Routine Immunization Program to Prevent Pneumococcal Pneumonia in Bangladesh

**DOI:** 10.7759/cureus.6082

**Published:** 2019-11-06

**Authors:** Sharmin K Luies, Md. Tarek Hossain, Haribondu Sarma

**Affiliations:** 1 Infectious Diseases Division, International Centre for Diarrhoeal Disease Research, Dhaka, BGD; 2 Maternal and Child Health Division, International Centre for Diarrhoeal Disease Research, Dhaka, BGD; 3 Nutrition and Clinical Services Division, International Centre for Diarrhoeal Disease Research, Dhaka, BGD

**Keywords:** awareness, bangladesh, parents, pcv, pneumonia, slums

## Abstract

Introduction

This study was conducted in two purposively selected slums of Dhaka to assess parents’ awareness of pneumococcal conjugate vaccine (PCV) in reducing the occurrence of death due to pneumococcal pneumonia.

Methods

Using a semi-structured questionnaire, face-to-face interviews were conducted with 150 parents. Data were analyzed using Statistical Package for the Social Sciences (SPSS), version 20 (IBM Corp., Armonk, NY) and Chi-square (χ2) test performed to determine associations.

Results

About 35% of parents were found to be aware of PCV; of them, 92.5% were informed by health service providers, and 81.1% mentioned benefits of PCV. Most parents were unaware of the PCV vaccination status of children, and no significant association was found between vaccination status and parents’ socioeconomic status. Aware parents vaccinated their children, and the association of awareness with vaccination status was statistically significant for PCV-1 (*P *= 0.04) and PCV-2 (*P *< 0.001). Although 7.4% of parents did not vaccinate their child with PCV-3 due to a child’s sickness or other priorities in household work, the association was significant (*P *= 0.01).

Conclusion

Strengthened efforts by health service providers, prioritizing dissemination of key messages on PCV, its benefits, and side-effects, can motivate parents and reduce dropout rates.

## Introduction

Bangladesh is one of the most densely populated countries in the world, with more than one thousand people living per square kilometer. About 37.2% of the population lives in urban areas [1], and the projection revealed that the urban population will be increased by 50% (from 54 million in 2015 to 81.4 million by 2029) [2]. The poor and marginalized people mostly live in urban slums and are deprived of access to basic services. Slums are usually defined as communities characterized by poverty, dense population, inadequate hygiene, sanitation, and basic services compared to neighboring non-poor areas [3,4]. Significant numbers of people are concentrated in Dhaka, Chattogram, and Khulna metropolitan areas that have 54% of the total urban population [5]. All major urban centers in the country have slums; 1.06 million people live in slums in the Dhaka division, followed by the Chattogram, Khulna and Rajshahi division, respectively [6].

Limited space in slum housing results in high population densities and poor living conditions; this overcrowding leads to a faster and wider spread of diseases [7]. Children living in impoverished communities suffer from substantial disease burden, and Pneumococcus was found consistently to be a leading cause of severe pneumonia, invasive disease, and death among those children. New vaccines against pneumococcal pneumonia provide much assurance to build momentum towards fighting against the effects of pneumonia, one of the leading causes of death among children below the age of five years worldwide [8]. In Bangladesh, an estimate revealed that the pneumococcal conjugate vaccine (PCV) could prevent an incredible one million episodes of pneumonia each year [9]. Considering the burden, the Government of Bangladesh (GoB) introduced PCV in the Expanded Programme on Immunization (EPI) in March 2015.

The EPI coverage evaluation survey showed that the fully-vaccinated children in Dhaka slums are only about 78% in 2016 [10]. Available literature demonstrated several factors that have an impact on the lower immunization coverage or dropouts in the slum areas of the country. Lack of information and knowledge, fear of the adverse effect or child’s sickness, lack of faith in the program, inconvenient service hours for working mothers, tendency of taking charges for vaccination among NGO service providers, rapid migration of the beneficiaries, and eviction of slums are highly influential factors [11-13]. Several studies assessed the immunization status of children at different times which revealed that social characteristics of individuals influence them to accept or seek out vaccinations [14-16]. Parents are the most common unit of analysis as decision-makers, a predictor of the acceptance of vaccination in terms of their education, affiliation to any social group, work status, etc. Introduction of any new vaccine into the community needs many efforts in disseminating the information to the mass people [17]. Considering the slum dwellers as a vulnerable group and the paucity of recent data, this study aims to explore the awareness among parents and their perceptions about the importance of PCV to immunize their children against pneumococcal pneumonia. This study also tried to identify the associated factors favoring the awareness and immunization coverage of this newly-introduced vaccine in the country.

## Materials and methods

Study design

This was a descriptive cross-sectional study aimed to determine the awareness among parents about the introduction of a new vaccine PCV in the urban slum areas of Dhaka city.

Study site and study population

This study was conducted in Dhaka City of Bangladesh. Two urban slums were selected purposively, one is Khilgaon slum area under Dhaka South City Corporation (DSCC) and another is the Korail slum area under Dhaka North City Corporation (DNCC). The study population comprised the parents of children aged 0-11 months.

Sampling strategy and sample

The sample-size was purposively determined to be 150 parents residing in the selected slums of Dhaka city considering time and limitation of resources to conduct this study; however, the sample was selected using a simple random sampling technique. An equal number of parents, 75 from Khilgaon slum and 75 from Korail slum, were chosen to have a comparison between the slums in respect of their geographic and socioeconomic indicators. A list of parents having children aged 0-11 months (155 at Khilgaon slum and 197 at Korail slum, totaling 352) was prepared for each slum. Using the list, sampling frames were developed for each slum by assigning a sequential number (1, 2, 3…n) to the eligible respondents. From the sampling frames, random numbers were generated electronically to get 150 parents (75 from each slum) to participate in the study.

Tools development

A semi-structured survey questionnaire consisting of open and closed-ended questions was developed to conduct this study. The survey questionnaire was thoroughly reviewed, and the investigators consulted the research supervisor and other experts before finalization. The study tool was pre-tested in another area (Sattola slum, Mohakhali) of Dhaka city, and necessary revision and amendment to the tool were ensured after the pre-test. Informed consent for the study was also produced and tested before starting the data collection.

Ethical approval and consent to participate

The Research Defence Committee of American International University-Bangladesh (AIUB), Dhaka, Bangladesh approved the study proposal (ethical approval reference number: 15-98603-2). In the field, all respondents provided with a consent form in detail about the study objectives, procedures, and the risks as well as the benefits involved. This information explained sufficiently to the respondents that they will be treated with full respect and dignity. There is an opportunity to discuss with the lead researcher if they required any further information and clarification. Written informed consent was obtained from each respondent before conducting the interviews. This study involved the non-invasive procedures and strictly adhered to ethical principles during the entire study process.

Data collection and field work

Data were collected from September to November 2016 from the parents during field visits, using a pre-tested, semi-structured and interviewer-administered questionnaire. To facilitate data collection and to have the necessary support from the parents and local authority, a letter containing permission was issued from the university authority. Convenient time of the respondent for the interview was considered during data collection, and second visits were made if deemed necessary.

Data were collected on socioeconomic and demographic variables, the number of parents aware of PCV, sources of the received information that include neighbors or service providers, or watching television, which makes the parents aware of the benefits and side-effects of PCV. The parents were asked to report about the vaccination center and distance from their residence to explore their awareness regarding the vaccination center available in their areas. Immunization status with PCV was cross-examined with the EPI card. The dose-specific eligibility of children for receiving PCV and the actual number of children who were vaccinated with PCV was also explored in this study. It was assumed that this information would provide the opportunity to have a better understanding of the awareness among the parents about PCV and to determine the association between awareness among the parents about PCV and vaccination status of their children.

Data analysis

All the data were entered in the pre-designed Microsoft Office Excel template, later imported to the statistical software called Statistical Package for the Social Sciences (SPSS), version 20 (IBM Corp., Armonk, NY). Of the total data, 5% were re-entered to control the quality of data-entry. Data were transferred to SPSS; cleaning and consistency checks were performed to prepare a clean dataset for analysis. The final dataset was analyzed using SPSS for cross-tabulation, and binary logistic regression (Chi-square test with significance level at *P *≤ 0.05, *P *= 0.000 means *P* < 0.0005) was performed to determine the association.

## Results

Characteristics of the respondents

A total of 150 parents (father or mother) participated in this study. An equal number of respondents were selected from two slums (n = 75) at Khilgaon and Korail of Dhaka city. Mostly females were available during the time of data collection, which was 86.0%, and only 14.0% was male; 42.7% of the respondents belonged to the age-group of 20-24 years. The number of family members was found to be in the range of 1-4 person(s) (56.0%). The majority of respondents (61.3%) were living in the slums for more than three years. One-third of the fathers (36.7%) and mothers (38.7%) had completed the primary level of education (Class 1-5). A good number of fathers were engaged as day-laborer (35.3%) and mothers were found to be homemakers (56.7%). 46.0% of the respondents' household income was in the range between 10,001-15,000 BDT, and some (10.0%) reported household income to be more than 15,001 BDT (Table 1).

**Table 1:** Socio-demographic characteristics of the respondents. PCV: Pneumococcal Conjugate Vaccine.

Characteristics		Aware of PCV		Total	P-value
		Yes	No		
		n (%)	n (%)	N (%)	
Sex					
	Male	7 (13.2)	14 (14.4)	21 (14.0)	0.83
	Female	46 (86.8)	83 (85.6)	129 (86.0)	
Age (years)					
	15-19	8 (15.1)	8 (8.2)	16 (10.7)	0.50
	20-24	22 (41.5)	42 (43.3)	64 (42.7)	
	25-29	16 (30.2)	28 (28.9)	44 (29.3)	
	≥30	7 (13.2)	19 (19.6)	26 (17.3)	
Number of household members					
	1-4 persons	30 (56.6)	54 (55.7)	84 (56.0)	0.80
	4-8 persons	19 (35.8)	38 (39.2)	57 (38.0)	
	>8 persons	4 (7.6)	5 (5.1)	9 (6.0)	
Duration of living					
	<1 year	9 (17.0)	13 (13.4)	22 (14.7)	0.67
	1-3 years	14 (26.4)	22 (22.7)	36 (24.0)	
	>3 years	30 (56.6)	62 (63.9)	92 (61.3)	
Educational status of fathers					
	No formal education	14 (26.4)	27 (27.8)	41 (27.3)	0.12
	Primary (Class 1-5)	14 (26.4)	41 (42.3)	55 (36.7)	
	Secondary (Class 6-10)	15 (28.3)	20 (20.6)	35 (23.3)	
	SSC passed	4 (7.6)	6 (6.2)	10 (6.7)	
	HSC passed	6 (11.3)	3 (3.1)	9 (6.0)	
Educational status of mothers					
	No formal education	14 (26.4)	25 (25.8)	39 (26.0)	0.15
	Primary (Class 1-5)	15 (28.3)	43 (44.3)	58 (38.7)	
	Secondary (Class 6-10)	18 (34.0)	26 (26.8)	44 (29.3)	
	SSC passed	2 (3.8)	1 (1.0)	3 (2.0)	
	HSC passed	4 (7.5)	2 (2.1)	6 (4.0)	
Occupation of fathers					
	Public/private service holder	15 (28.3)	21 (21.6)	36 (24.0)	0.80
	Business/petty business	14 (26.4)	22 (22.7)	36 (24.0)	
	Day-laborer	16 (30.2)	37 (38.1)	53 (35.3)	
	CNG/Auto-driver	7 (13.2)	15 (15.5)	22 (14.7)	
	Unemployed	1 (1.9)	2 (2.1)	3 (2.0)	
Occupation of mothers					
	Public/private service holder	9 (17.0)	23 (23.7)	32 (21.3)	0.53
	Homemaker	33 (62.3)	52 (53.6)	85 (56.7)	
	Day-laborer/Maidservant	11 (20.7)	22 (22.7)	33 (22.0)	
Household income (monthly)					
	Tk 5000-10000	21 (39.6)	45 (46.4)	66 (44.0)	0.40
	Tk 10001-15000	24 (45.3)	45 (46.4)	69 (46.0)	
	Tk > 15001	8 (15.1)	7 (7.2)	15 (10.0)	

Awareness among parents about PCV and vaccination status of children

Two-thirds of the respondents were not aware (64.7%) of PCV, and the rate was comparatively higher among the parents residing in Korail area (68.0%). Those who were aware of PCV mostly received information from the health service providers (92.4%). A significant number of the respondents (81.1%) could mention the actual benefit of PCV; however, they were mostly (62.3%) unaware of the side-effects of this vaccine. Vaccination center was mostly (64.0%) EPI outreach sites that are satellitic in nature and located at a distance of less than one kilometer from the household (86.0%). Majority of the respondents (66.6%) could not be able to mention the immunization status of their children with PCV. Those who were able to report the status were mostly motivated by the service providers (53.5%), followed by their own urges to prevent their children from pneumonia (37.2%). Almost all the respondents (n = 144, 99.3%) reported the possession of an EPI card, and most of them (n = 111, 77.1%) could show the EPI card during data collection (data not shown in the table). Three doses of PCV are scheduled according to the age of the child, which is six weeks for PCV-1, 10 weeks for PCV-2, and 18 weeks for PCV-3. Cross-verification of vaccination status with PCV following the EPI card revealed that 94.2% of children received PCV-1, 86.7% received PCV-2, and 76.0% received PCV-3 (Table 2).

**Table 2:** Awareness of PCV among parents and the vaccination status of children with PCV. ^a^ Clinics operated by the Government or NGOs provide immunization service with other health services. ^b^ Usually held in the houses of slum-dwellers or such a suitable place near the slum. ^c^ Showed EPI card. PCV: Pneumococcal Conjugate Vaccine; EPI: Expanded Programme on Immunization.

Characteristics		Khilgaon	Korail	Total	P-value
		n (%)	n (%)	N (%)	
Aware of PCV (N = 150)					
	Yes	29 (38.7)	24 (32.0)	53 (35.3)	0.39
	No	46 (61.3)	51 (68.0)	97 (64.7)	
Source of information (N = 53)					
	Neighbors	1 (3.4)	2 (8.3)	3 (5.7)	0.39
	Television	--	1 (4.2)	1 (1.9)	
	Health service providers	28 (96.6)	21 (87.5)	49 (92.4)	
Aware of the benefits of introducing PCV (N = 53)					
	Vaccinate against pneumonia	23 (79.3)	20 (83.3)	43 (81.1)	0.17
	Strengthen communication with health service providers	--	3 (12.5)	3 (5.7)	
	Do not know	6 (20.7)	1 (4.2)	7 (13.2)	
Aware of side-effects of PCV (N = 53)					
	Fever	4 (13.8)	5 (20.8)	9 (17.0)	0.07
	Rash	3 (10.3)	5 (20.8)	8 (15.0)	
	No side-effects	--	3 (12.5)	3 (5.7)	
	Do not know	22 (75.9)	11 (45.9)	33 (62.3)	
Type of vaccination center (N = 150)					
	Static clinic^a^	47 (62.7)	7 (9.3)	54 (36.0)	<0.001
	EPI outreach site^b^	28 (37.3)	68 (90.7)	96 (64.0)	
Distance of EPI center from residence (N = 150)					
	<1 km	67 (89.3)	62 (82.7)	129 (86.0)	0.23
	1-3 km	8 (10.7)	13 (17.3)	21 (14.0)	
Reported PCV vaccination status (N = 150)					
	Yes	21 (28.0)	22 (29.3)	43 (28.7)	0.02
	No	7 (9.3)	--	7 (4.7)	
	Do not know	47 (62.7)	53 (70.7)	100 (66.6)	
Motivations for PCV vaccination (N = 43)					
	Prevent from pneumonia	4 (19.0)	12 (54.5)	16 (37.2)	0.04
	Prevent from diseases	2 (9.5)	2 (9.1)	4 (9.3)	
	Motivated by health service provider	15 (71.5)	8 (36.4)	23 (53.5)	
PCV vaccination status verified with EPI card (N = 111)^c^					
Eligible for PCV-1					
	Yes	49 (94.2)	55 (93.2)	104 (93.7)	0.82
	No	3 (5.8)	4 (6.8)	7 (6.3)	
Vaccination status of PCV-1 (N = 104)					
	Yes	46 (93.9)	52 (94.5)	98 (94.2)	0.88
	No	3 (6.1)	3 (5.5)	6 (5.8)	
Eligible for PCV-2					
	Yes	45 (86.5)	45 (76.3)	90 (81.1)	0.16
	No	7 (13.5)	14 (23.7)	21 (18.9)	
Vaccination status of PCV-2 (N = 90)					
	Yes	41 (91.1)	37 (82.2)	78 (86.7)	0.21
	No	4 (8.9)	8 (17.8)	12 (13.3)	
Eligible for PCV-3					
	Yes	41 (78.8)	34 (57.6)	75 (67.6)	0.01
	No	11 (21.2)	25 (42.4)	36 (32.4)	
Vaccination status of PCV-3 (N = 75)					
	Yes	34 (82.9)	23 (67.6)	57 (76.0)	0.12
	No	7 (17.1)	11 (32.4)	18 (24.0)	

Association of parents’ awareness of PCV by selected indicators

Using selected socio-demographic indicators, such as sex, age, number of household members, occupation, and household income, etc., no significant association was found with the awareness of PCV among parents and the vaccination status. There was no significant association between awareness of PCV with father’s and mother’s education level (*P* = 0.12 and *P *= 0.15, respectively). Significant numbers of fathers and mothers with formal education (Class 1-10) were not aware of PCV compared to those who had no formal education. Higher percentage of children whose mothers were homemakers (56.7%) were aware of PCV compared to those who were engaged with income-generating activities (Table 1). Type of vaccination center had an influence (*P* < 0.001) as the EPI outreach sites bring services to the doorsteps of slum population. Awareness of PCV showed association in reporting vaccination status (*P* = 0.02) of their children (Table 2).

Association between awareness among parents about PCV and immunization with PCV (based on the eligibility of children and availability of EPI card) was assessed in this study and was found statistically significant. In the case of PCV-1 and PCV-2, all the parents who were aware of PCV were found to vaccinate their children with this vaccine, and these associations were found statistically significant (*P *= 0.04 and *P *< 0.001, respectively). About 7.4% of parents did not vaccinate their children with PCV-3, despite their awareness of PCV. This association was also found significant with a *P*-value of 0.01 (Table 3).

**Table 3:** Association between awareness among parents about PCV and vaccination status of children with PCV†. ^†^ The analysis was done based on eligibility of the children in receiving PCV-1, PCV-2 and PCV-3 dose verified through the EPI card. PCV: Pneumococcal Conjugate Vaccine; EPI: Expanded Programme on Immunization.

Characteristics		Aware of PCV			P-value
		Yes	No	Total	
		n (%)	n (%)	N (%)	
Vaccination status of PCV-1 (N = 104)					
	Yes	41 (100.0)	57 (90.5)	98 (94.2)	0.04
	No	0.0	6 (9.5)	6 (5.8)	
Vaccination status of PCV-2 (N = 90)					
	Yes	36 (100.0)	42 (77.8)	78 (86.7)	<0.001
	No	0.0	12 (22.2)	12 (13.3)	
Vaccination status of PCV-3 (N = 75)					
	Yes	25 (92.6)	32 (66.7)	57 (76.0)	0.01
	No	2 (7.4)	16 (33.3)	18 (24.0)	

## Discussion

This study demonstrates how awareness among parents about the PCV in routine immunization program influence the vaccination status of their children. We documented a number of issues that demonstrated the association between parental awareness of PCV and vaccination status of children with PCV. Only 35.3% were found aware of PCV, and a significant number of respondents (66.6%) failed to mention the vaccination status of their children with PCV. A similar type of study in India found about 36% of mothers to be aware of the newly-introduced pentavalent vaccine, and only 6% of mothers could mention the name of diseases prevented by this vaccine [18]. Findings from this study revealed that a good number of parents without having formal education and with low socioeconomic status are aware of PCV as informed by the health service providers. They had easy access to immunization services at their doorsteps mostly provided by the EPI outreach site. This was in contrast to the findings of other studies conducted in India, Nepal, and Uganda, which found that education and socioeconomic status were significant over independent predictors of immunization status [19-22]. In Pakistan, the mother’s illiteracy has been identified as one of the major factors that hinder the success of the immunization program [23]. Also, in Sri Lanka, the findings demonstrated that maternal knowledge on immunization has an influence on the vaccination status of the children, and these findings are in agreement with the findings of the present study [24]. An interventional study revealed that improved social mobilization activities help in progressing to vaccination status in Bangladesh, regardless of education or occupation status of the parents [25].

Findings from other studies showed that superficial knowledge of the schedule and inadequate identification of proper motivation in the target population for completing the schedule led to a large proportion of the children being partially immunized, although a majority of the population recognized the importance of immunization [26]. In this study, a significant association was found between awareness among parents about PCV and the vaccination status of children with PCV, following the EPI card. In the case of PCV-1 and PCV-2, all aware parents vaccinated their children upon their eligibility for receiving the vaccine according to the age of their children. Despite awareness of PCV, a few numbers of parents (7.4%) did not vaccinate their children with PCV-3 due to a child’s sickness or other priorities in household work. It is apparent that the third dose of PCV was lagging compared to the previous two doses, which is also reported in the post-introduction evaluation (PIE) conducted in November 2015. The PIE team recommended ensuring close monitoring of the coverage of PCV-3 to investigate the causes, especially in the Dhaka City Corporations and exploring options for improvements [27]. Previous evidence showed that more than 30% of the partial immunization or no immunization occurs due to a lack of awareness [28,18]. Moreover, the mother had the knowledge gap in the immunization schedule and the number of doses [24]. The findings of this study also demonstrated that a good number of respondents failed to mention the vaccination status of their children with PCV, which reflects their inadequate awareness of the vaccination schedule. Therefore, more investigation is needed on the reasons, especially in the slum areas where mothers are found having less aware of specific vaccines and preventable diseases; we need to identify the pathway to reach those dropouts.

Community trust towards the health service providers and effective social mobilization created a strong demand for immunization services, with rapid adaptation to the new immunization schedule [29,27]. This is also reflected in this study as health service providers have been mentioned by 92.4% of aware respondents as sources of information on PCV. Significant determinants of PCV uptake revealed in this community included awareness of PCV, lack of knowledge on diseases preventable by PCV, benefit and side-effects of the vaccine, and work-related reasons hindering parents from taking the child for immunization. These results indicate important areas for intervention to improve immunization uptake among the slum areas in Dhaka city. Although the availability of service centers and information from the health service providers have been reported in this study, the awareness of PCV was found low in the selected areas of this study. Health service providers should use innovative approaches (e.g., pictorial education intervention) to disseminate information on the vaccine, including its benefits and proper guidance on possible side-effects to the parents, which can possibly reduce the dropout rates in vaccination [26,30]. This study found that having awareness about a particular vaccine has an implication on the vaccination status, which may be applicable to other vaccines as well. The results depending on this sample size cannot be generalized and representative to the community. Since this study was conducted among a definite population at a particular point of time, therefore, assessment of PCV coverage of all three doses per child is beyond the scope of this study. Only associations of parental awareness with vaccination status with PCV can be reported but not the causal effects.

## Conclusions

The present study demonstrated that awareness among parents about PCV in the routine immunization program has a significant influence on the vaccination status of their children. Appropriate programmatic support, wide acceptability of vaccinations, availability, and accessibility to vaccination center have a positive influence on the vaccination status of children. Awareness of PCV is low in the selected areas that hinder the full immunization coverage in the study areas. Key messages provided by the health service providers should be enhanced, and the use of innovative approaches for PCV administration, including dissemination of messages on its benefits and proper guidance on possible side-effects to the parents could possibly reduce the dropout rates. However, studies with more robust methodology need to be designed to identify factors associated with complete immunization, in order to design better interventions for the slum areas in Bangladesh.
